# Apocrine Carcinoma of Breast: A Case Report with Review of the Literature

**DOI:** 10.1155/2013/170918

**Published:** 2013-06-24

**Authors:** Jyotsna V. Wader, Akash Jain, Suresh J. Bhosale, Pandurang G. Chougale, Sujata S. Kumbhar

**Affiliations:** ^1^Department of Pathology, Krishna Institute of Medical Sciences, Karad, Maharashtra 415110, India; ^2^Department of Surgery, Krishna Institute of Medical Sciences, Karad, Maharashtra 415110, India

## Abstract

Apocrine carcinoma is a very rare form of breast malignancy with an incidence of <1% of female invasive breast carcinoma. We report a case of apocrine carcinoma in a 42-year female with marked adenosis showing apocrine metaplasia and discuss the criteria to diagnose apocrine carcinoma with the emerging concept of androgen receptor positivity with its implication on treatment and management of the patient.

## 1. Introduction

Apocrine carcinoma (AC) is a very rare form of breast malignancy with an incidence of <1% of female invasive breast carcinoma [1], with sparse information available in the literature [2]. Microscopically, apocrine carcinoma demonstrates the same architectural growth pattern as invasive ductal carcinoma, not otherwise specified type (IDC-NOS), differing only in their cytological appearance. Cells are characterized by typical apocrine features, namely, abundant eosinophilic granular cytoplasm and prominent/multiple nucleoli [2]. According to emerging evidence, apocrine carcinomas tend to show estrogen and progesterone receptor negativity and androgen receptor positivity (ER−/PR−/AR+); and expression of Gross cystic disease protein fluid-15 (GCDPF-15) [3, 4]. Apocrine carcinomas show a unique response to androgen (fluoxymesterone) administration as a part of treatment [4]. We report a case of invasive apocrine carcinoma of breast as it is a very rare morphological entity.

## 2. Case-Report

A 42-year-old female presented to Surgical OPD of our hospital, with history of lump in the upper inner quadrant of her right breast, for which lumpectomy was done outside and sent for histopathological examination, one month back, histopathology report was given as Invasive ductal carcinoma. There was no history of any nipple retraction and discharge, no significant past history of tuberculosis, and so forth. The patient was afebrile with no abnormality detected on general physical examination. Local examination of the right breast showed a linear scar mark, medial to the nipple. The breast was tender to touch while no mass lesion was felt. Left breast was unremarkable with no axillary lymphadenopathy. In view of the histopathology report, right-sided modified radical mastectomy (MRM) was performed at our hospital and excised specimen was sent for histopathological examination.

Right MRM specimen measuring 17 × 16.5 × 3 cm and weighing 500 gms was grossed. Overlying skin showed a scar mark. Serial cut section revealed a cavity (postlumpectomy); however, no any residual tumor could be appreciated on gross examination, representative bits were processed, and H&E stained tissue sections were obtained. Outside slide and blocks reported as invasive ductal carcinoma were also reviewed.

**Figure 1:**
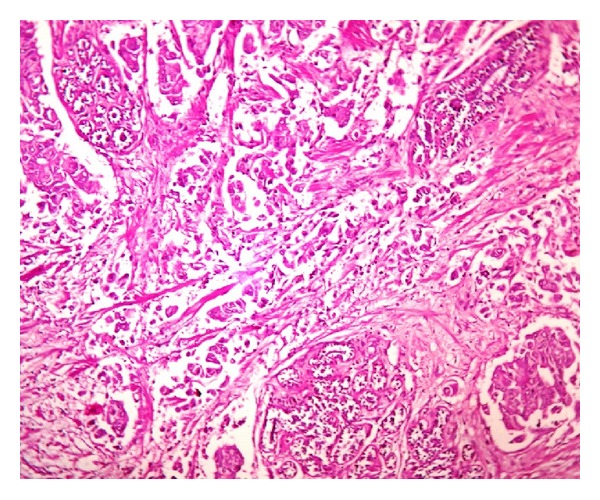
Photomicrograph showing tumor along with foci of adenosis showing apocrine metaplasia (H&E: 10x).

**Figure 2:**
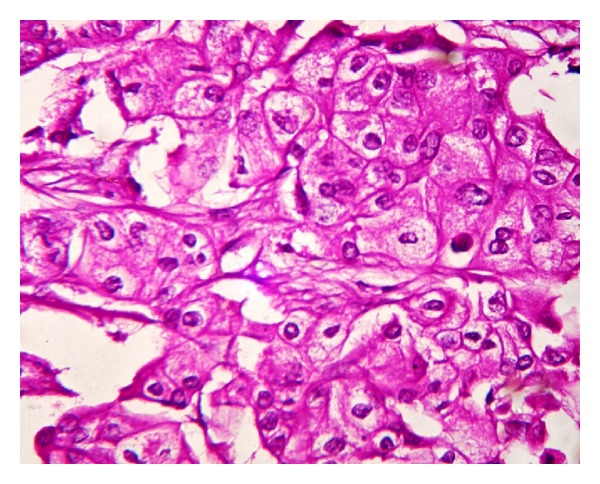
Photomicrograph showing large tumor cells having abundant granular eosinophilic cytoplasm and large round nuclei (H&E: 40x).

On light microscopy, outside slides (from lump) showed a tumor composed of large cells arranged in tubules, glandular pattern, and sheets, having abundant eosinophilic granular cytoplasm with distinct cell margins, large round nuclei with vesicular chromatin pattern, and occasional prominent nucleoli. Tumor cells showed PAS positivity with diastase resistance. Bloom Richardson score was 2 + 2 + 1 = 5 (Grade I). Also noted was marked adenosis with apocrine metaplasia in the adjacent breast tissue (Figures 1 and 2). 

Sections from MRM specimen revealed minimal residual tumor with surrounding breast tissue showing marked adenosis with apocrine metaplasia. Also noted was foreign body giant-cell reaction in the overlying skin—consistent with prior history of surgery (lumpectomy). Final diagnosis was given as invasive apocrine carcinoma, right breast, with minimal residual tumor in MRM specimen. All lymph nodes, surgical margins, skin, nipple, and areola were free from tumor. Immunohistochemistry was done which showed estrogen and progesterone receptor negative, androgen receptor positive (ER−/PR−/AR+).

## 3. Discussion

Division of mammary cancer into various histologic types has been of interest to pathologists for many years [5]. Apocrine metaplasia characterized by finely granular, pale eosinophilic cytoplasm, and a tendency to apical budding of the cytoplasm is generally regarded as an indicator of low potential for a given lesion undergoing malignant transformation. The malignant transformation of this apocrine epithelium was first described by Krompecher in 1916 [5, 6].

The incidence of infiltrating apocrine carcinoma is unclear, as the definition and consequently the reported incidence vary considerably. Gayatri et al. included this entity under the group of “relatively rare carcinomas” [1]. Azzopardi reported an incidence of apocrine carcinoma between 0.3 and 0.4% of all breast carcinomas, Frable and Key reported 1%, Bonser et al. reported 14.5%, and Haagensen reported 62% [1, 4].

This highly variable incidence confirms the need for standardized criteria for the diagnosis of apocrine carcinoma. Japaze et al., 2005, proposed that criteria are as follows: (1) apocrine features consisting of 75% of cells, (2) large cells with eosinophilic granular cytoplasm, (3) nucleus to cytoplasmic ratio of 1 : 2 or more, (4) nucleus large, round, and vesicular may be pleomorphic, (5) Sharply defined borders. Minor and nonmandatory criteria include prominent nucleoli in >50% of fields and apical cytoplasmic snouts into luminal spaces [1]. Our case fulfilled all the five criteria.

Wald and Kakulas have reviewed various histogenetic theories to account for the origin of apocrine-like epithelium in the breast. The relation of this epithelium to true apocrine glands is only a morphological similarity. Dawson has denied the origin of this epithelium from apocrine glands [5].

Apocrine carcinoma always shows moderate to marked nuclear pleomorphism and tubule formation is rarely greater than 75%. Mitotic count is variable (1–3). Therefore, most apocrine carcinomas are modified Scarff-Bloom-Richardson grade 2 or 3 [4]. However, our case belonged to grade 1, as she had moderate nuclear pleomorphism and low mitotic count.

Ongoing studies about apocrine carcinomas revealed gross cystic disease fluid protein-15 (GCDFP-15) positivity on molecular analysis [3, 4]. Hormonal status of apocrine carcinomas is found to be basal-like triple negative breast cancer with androgen receptor positivity [3]. Hormonal profile in our case was consistent with it being ER−/PR−/AR+.

Apocrine carcinoma has a prognosis similar to IDC-NOS-type breast cancer, when matched for stage and grade [2]. Durham and Fechner [4] in 2000 discussed that one may question why apocrine carcinoma should be classified as a separate group if there are neither diagnostic nor prognostic differences between nonapocrine infiltrating adenocarcinomas and apocrine carcinomas. However, there seems to be a potential unique response to androgen (fluoxymesterone) administration as a part of treatment; that may justify identifying apocrine carcinoma as an entity different from usual ductal carcinoma, which was further emphasized by Tsutsumi [3], 2012, by demonstrating androgen receptor positivity, which may lead to different clinical behavior and management protocols. Based on this, our patient is put on hormonal therapy including androgen analogue along with supportive care. 

## 4. Conclusion

Apocrine carcinoma is a rare and distinct morphological type of invasive breast cancer. Although prognostically same as IDC-NOS, apocrine carcinoma should be diagnosed as separate entity, as there are growing bodies of evidence that apocrine carcinoma may have different hormonal profile and may show different clinical behavior with a unique response to androgens.
